# Evaluating battery electric vehicle usage in the EU: A comparative study based on member state energy mixes

**DOI:** 10.1016/j.heliyon.2024.e30655

**Published:** 2024-05-03

**Authors:** Denes Kocsis, Judit T. Kiss, Istvan W. Arpad

**Affiliations:** a Department of Environmental Engineering, Faculty of Engineering, University of Debrecen, 4032, Debrecen, Hungary; b Department of Engineering Management and Enterprise, Faculty of Engineering, University of Debrecen, 4032, Debrecen, Hungary; c Department of Mechanical Engineering, Faculty of Engineering, University of Debrecen, 4032, Debrecen, Hungary

**Keywords:** Carbon emission, Energy efficiency, Electric vehicle, Energy conversion

## Abstract

The transport sector is undergoing a major transformation, as battery electric vehicles (BEV) are gaining ground. Therefore, assessing the sustainability aspects of their use is crucial to obtaining a clear picture of the sector. This article aims to meet this requirement by using European Union (EU) data for the period 2011 to 2021 and focuses not only on EU-27 aggregates but also on each member state separately. For the evaluation, a well-to-wheel (WTW) method was used to calculate two parameters: energy-specific CO_2_ emissions (ε) and total efficiency of energy conversions, transmission, and battery (η_total_). For these values, the annual electricity mixes of the countries were tracked in 5 + 1 categories (combined cycle gas turbine (CCGT), thermal power plant, biofuels, nuclear power plant (NPP), renewables, and imports). The calculated results were illustrated by sustainability matrices describing the former and current positions of the countries. The EU-27 aggregate achieved a 0.04 increase (from 0.37 to 0.41) in total efficiency and a 29 gCO_2_/MJ_motion_ decrease (from 113 to 84 gCO_2_/MJ_motion_) during the period. This ε value for 2021 was around half the world average. However, very significant differences were identified between member states, which are also assessed in the article with special emphases on the five most populated EU countries (Germany, France, Italy, Spain, and Poland).

## 1 Introduction

The efforts focusing on the reduction of carbon dioxide (CO_2_) emissions strongly affect the transportation sector, pushing it towards more sustainable solutions. Battery electric vehicles are promising in terms of CO_2_ reduction and enhancing energy conversion efficiencies.

Regarding CO_2_ emissions, it is clear that there is a great difference between internal combustion engine vehicles (ICEV) and battery electric vehicles during their usage [1]. This has also been proven by real-world driving investigations [2,3]. A crucial factor in the CO_2_ emissions of electric vehicles (EV) is the electricity production mix which is used to charge the batteries. Kucukvar et al. [4]. investigated the environmental efficiency of electric vehicles with various future electricity mix scenarios in Europe. They concluded that clean energy sources have a key role in bringing carbon neutral and circular economy opportunities in the transport sector. Koroma et al. [5] examined the environmental impacts of a BEV charged with an average EU electricity mix. They also made future projections, anticipating changes in the mix. Shafique et al. [1] also highlighted the importance of the electricity mix in their study examining the situation in Hong Kong, where the mix of 2019 is not suitable for electric vehicles, but according to their expectations the situation in 2050 will be different due to the increasing ratio of renewable energy sources. Petrauskiene et al. [6] published a comparative study between electric and conventional vehicles and also made forecasts until 2050 based on Lithuanian data. They also quantified the importance of the electricity sources in their forecasts. Similarly, Joshi et al. [7] pointed out the importance of a low-carbon intensity mix and described the effect on reducing the emissions of BEVs. Major events in the recent past, such as the outbreak of the Russia-Ukraine war which have resulted in a significantly altered gas situation for many parts of the world, including Europe, create the necessity to rethink expectations regarding the future electricity mix. Ghisellini et al. [8] assessed multiple scenarios for Italy and Europe in their article, taking into consideration the changed circumstances. Scarlat et al. [9] examined European countries in terms of the emission of their electricity production and usage and they revealed significant variations between countries. At the 28th Conference of the Parties (COP28), fast-tracking the energy transition was one of the four priority areas under the presidency's plan of action. Besides the many recent scientific research studies, there are still major research gaps in the sustainability assessment of electric vehicles [10], which includes the changes in their CO_2_ emissions during operations.

The comparison of vehicles with different powertrains is a prominent research topic, as much recent scientific research focuses on it. Well-to-wheel is a widespread technique used for evaluation, and it is described as the only fair way to compare vehicles with different powertrains [11]. The BEV usage within the European Union is also usually analyzed by WTW studies. These publications mostly focus on EU-27 values, like Rovai et al. [12], where a comparison was made involving China, the USA, the EU and Brazil. Bíró and Kiss [13] investigated various heavy-duty vehicle propulsions by WTW, and they also highlighted the importance of the electricity mix and pointed out the differences between European countries. There are also investigations based on national data, such as the work of Garcia et al. [14], where Spanish data was used to evaluate the carbon footprint of BEVs considering average and marginal electricity mixes. Clemens and Clemens [15] investigated different scenarios to decarbonize the energy consumption of Austria and they examined BEV efficiency. Jochem et al. [16] conducted an investigation to forecast the future situation in Germany by assessing the CO_2_ emissions of electric vehicles.

The number of new BEV passenger cars has increased rapidly since 2010. According to the European Environment Agency [17], new registrations in Europe were less than 600 in 2010 and increased to more than seven thousand in 2011. Therefore, 2011 was chosen as the starting year of our investigation. The last examined year (2021) had more than 876,000 new BEV registrations [17]. According to the International Energy Agency's [18] predictions for 2030, more than 40 million electric cars may be sold globally, six times more than in 2021. Currently, China, the European Union and the United States account for 90 % of electric car sales, but this is projected to fall just over 60 % by 2030 [18]. These circumstances taken together highlight the intensity of changes, which elevates the importance of investigations focusing on the challenges related to electromobility.

The impact of BEV usage on energy consumption is also an important issue, which needs further research [19]. It should also be noted that BEV sales forecasts for the next few decades strongly depend on parameters that cannot be accurately determined. Such parameters are BEV subsidies by governments and the consumer's willingness to pay more for environment-friendly technological solutions [[20], [21], [22], [23]]. Furthermore, events such as the Russia-Ukraine conflict mentioned above can also have an influence on BEV sales, which was investigated by Wei et al. [24] in the case of China.

In this study, BEV usage was evaluated by a WTW method from a sustainability perspective, represented by total energy conversion efficiencies and CO_2_ emissions. This article covers a relatively long time interval (from 2011 until 2021), examining the overall EU-27 situation and all member states individually. Therefore, this article aimed to provide the same base comparison of all EU countries, analyzing the outcomes of their efforts to decarbonize and move towards more sustainable mobility.

## 2 Materials and methods

**Fig. 1:**
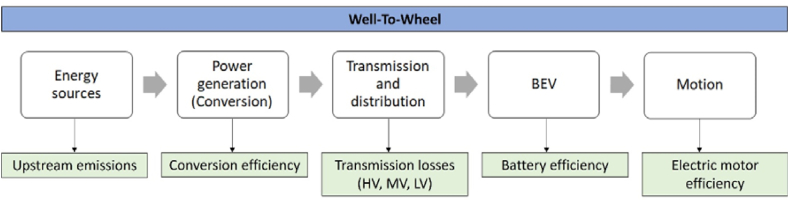
WTW approach in the case of BEVs (authors' creation).

A WTW investigation of BEV usage was carried out to evaluate EU countries' former and current situation from a sustainability perspective. WTW methodology, as a simplified life cycle assessment (LCA), examines the CO_2_ emissions originating from the production, transportation, and distribution of fuel (well-to-tank (WTT)) in addition to the evaluation of the powertrain's efficiency. Fig. 1 illustrates the boundaries of the investigation.

Twenty-seven member states of the EU were involved, with the time range from 2011 until 2021. For all years, the electricity mix of the member states and the EU-27 were generated based on Eurostat data [[25], [26], [27]] with the following main categories: CCGT (natural gas (NG)), TPP (thermal power plant), biofuels, NPP, renewables (including hydro, geothermal, wind, solar and tide) and imports. The two following parameters were used for the evaluation:

• η_total_: total efficiency of energy conversions, transmission, and battery.

(1) $$\eta_{total}=\prod_{i}\eta_{i}=\prod_{i}\frac{E_{out,i}}{E_{in,i}}\;\left[-\right]\text{,}$$

where in equation (1) η_i_ is the ith step of energy conversion, transmission or storage determined by the ratio of the energy output ($E_{out,i}$) and input ($E_{in,i}$). In this investigation, according to Fig. 1, the efficiencies listed in Table 1 were taken into consideration.

• ε: energy-specific CO_2_ emission parameter for BEV.

(2) $$\varepsilon =\frac{mass\;of\;{CO}_{2}eq\;produced\;upstream\;\&\;electricity\;generation}{final\;energy\;of\;motion\;of\;vehicle}*\frac{1}{\eta_{tr}*\eta_{b}*\eta_{em}}\;\left[\frac{g_{{CO}_{2}}}{{MJ}_{motion}}\right]$$

**Table 1:** Efficiencies involved in the calculation [−].

Power generation	Transmission	Battery	Electric motor
η_pg_	η_tr_	η_b_	η_em_

Similar parameters have already been used by Arpad et al. [28] for ICEV and EV, but here (in equation (2)) ε is extended with upstream emissions, transmission losses, battery efficiency and electric motor efficiency. These were also taken into consideration for covering a wider range of the WTW analyses.

### 2.1 Upstream emissions

Upstream emissions involve all emissions up to the power plant gate, i.e. the extraction, refining and transportation of the fuel. Data from the Joint Research Centre WTT report [29] was used for the calculation, and the applied values can be seen in Table 2.

**Table 2:** Upstream emission values [gCO_2eq_/MJ] [29].

Brown coal	Natural Gas	Solid biofuels	Liquid biofuels	Biogases	Nuclear
1.7	12.8	0.7	46.8	14.9	1.4

For the TPP category, brown coal's upstream emission value was always used as representing far the largest share of all combustion fuels in the EU, as natural gas and biofuels are taken into consideration in separate groups (CCGT and biofuels) within the electricity mix.

In the case of biofuels, the role of the three subcategories (solid biofuels, liquid biofuels, and biogases) was calculated, as it showed a vastly different picture in numerous member states. For example, in 2011 the solid biofuels ratio was around 98 % in Estonia, while it was 0 % in Greece which had 100 % of biofuels in the group of biogases. The ratio of the three subgroups within biofuels was tracked for all examined years, and the biofuels annual upstream emissions were weighted by the ratio of each subcategory.

### 2.2 Power generation

The power generation's (conversion's) efficiency depends on the plant type. For every year in each category the same efficiency was used, summarized in Table 3.

**Table 3:** Power generation efficiency (or sustainability effectiveness in case of biofuels and renewables) η_pg_ [−] [28].

CCGT (NG)	TPP^a^	Biofuels^b^	NP	Renewables^b^	Import^c^
0.55	0.5	1	0.33	1	EU-27

a Efficiency with heat generation was used for the calculation.

b Renewables' and biofuels' effectiveness is 1 as the sources never run out.

c The current year's weighted average value of the EU-27 production was applied for the efficiency of import.

The energy-specific CO_2_ emission of the electricity production can be obtained based on the efficiencies. In this manuscript, the values calculated by Árpád et al. [28] were used: CCGT 139 gCO_2_/MJ; TPP 186 gCO_2_/MJ; and 0 gCO_2_/MJ for the biofuels, NPP and renewables.

### 2.3 Transmission, battery and electric motor efficiencies

Overall transmission losses for High Voltage (HV), Medium Voltage (MV), and Low Voltage (LV) systems are assumed to be 7 %, based on literature data for the EU member states [29]. Therefore η_tr_ = 0.93 was used in all cases.

Lithium-ion batteries used in EVs can have very high efficiency [[30], [31], [32]]. A uniform value of 0.9 was applied to the parameter η_b_.

The efficiency of electric motors is significantly higher than that of internal combustion engines. During the calculations, 0.9 was used for η_em_, while there may already be better efficiencies than this [33].

### 2.4 Calculation of energy-specific CO_2_ emissions

**Fig. 2:**
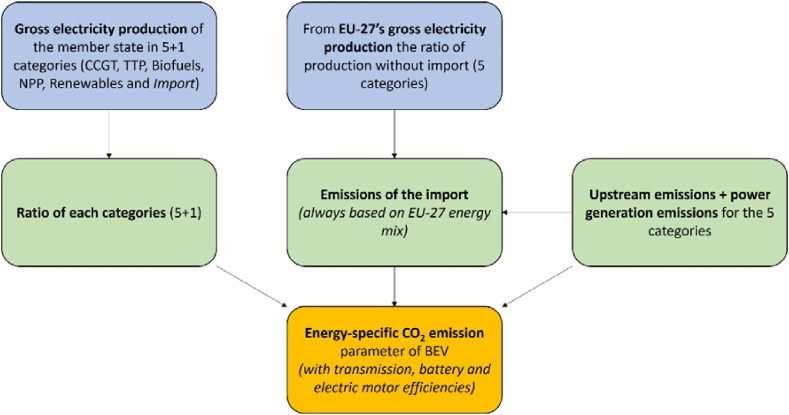
Calculation procedure of energy-specific CO_2_ emissions (authors' creation).

The starting point of the calculation was the annual gross electricity production of the country in the mentioned 5 + 1 categories (CCGT (NG), TPP, biofuels, NPP, renewables and imports). The ratio of each category (5 + 1) was calculated from the gross electricity production. The current year's gross electricity production ratios of the EU-27 were applied to handle the imports. This was used uniformly for all member states with a positive import balance in the examined year. Upstream and power generation emissions were summarized for the 5 + 1 category and corrected by the transmission, battery, and electric motor efficiencies. By this calculation process, the energy-specific CO_2_ emissions were obtained for all countries (plus the EU-27) in all examined years (2011–2021). The calculation procedure is summarized in Fig. 2.

## 3 Results and discussion

### 3.1 Electricity mix

The electricity mix of the EU-27 has undergone significant changes over the period under review. Among many interesting components, it is noteworthy that the amount of energy produced in the EU has not changed as significantly as certain subcategories within the total amount produced. From the current WTW analysis, the continuous increase in the case of renewable sources and biofuels clearly points towards lower specific CO_2_ emissions. TPP and NPP showed a considerable decrease, while the imports to the EU were insignificant (less than 0.6 %) during the period. Table 4 shows the gross electricity production of the EU-27. The same tables were prepared for all member states.

**Table 4:** Gross electricity production [TWh] and percentages for the EU-27, 2011–2021 [[25], [26], [27]].

	CCGT	TPP	Biofuels	NPP	Renewables	Import	Total
2011	558.2	870.2	119.0	837.8	551.9	1.0	**2938**
	*19*.*0 %*	*29*.*6 %*	*4*.*1 %*	*28*.*5 %*	*18*.*8 %*	*0*.*0 %*	
2012	484.1	881.9	132.9	812.0	623.4	6.8	**2941**
	*16*.*5 %*	*30*.*0 %*	*4*.*5 %*	*27*.*6 %*	*21*.*2 %*	*0*.*2 %*	
2013	415.1	859.2	139.2	806.2	696.7	0.0	**2916**
	*14*.*2 %*	*29*.*5 %*	*4*.*8 %*	*27*.*6 %*	*23*.*9 %*	*0*.*0 %*	
2014	357.0	820.7	144.3	812.6	721.9	0.0	**2857**
	*12*.*5 %*	*28*.*7 %*	*5*.*1 %*	*28*.*4 %*	*25*.*3 %*	*0*.*0 %*	
2015	396.3	833.8	149.4	786.7	734.4	0.0	**2901**
	*13*.*7 %*	*28*.*7 %*	*5*.*2 %*	*27*.*1 %*	*25*.*3 %*	*0*.*0 %*	
2016	466.2	788.9	151.1	768.0	747.8	0.6	**2923**
	*16*.*0 %*	*27*.*0 %*	*5*.*2 %*	*26*.*3 %*	*25*.*6 %*	*0*.*0 %*	
2017	525.2	766.4	153.6	759.4	749.9	0.0	**2955**
	*17*.*8 %*	*25*.*9 %*	*5*.*2 %*	*25*.*7 %*	*25*.*4 %*	*0*.*0 %*	
2018	490.7	718.8	155.5	761.9	811.1	8.9	**2947**
	*16.6 %*	*24*.*4 %*	*5*.*3 %*	*25*.*9 %*	*27*.*5 %*	*0*.*3 %*	
2019	569.3	564.2	159.7	765.3	843.9	2.9	**2905**
	*19*.*6 %*	*19*.*4 %*	*5*.*5 %*	*26*.*3 %*	*29.1 %*	*0*.*1 %*	
2020	561.0	452.1	162.6	683.5	925.6	14.0	**2799**
	*20*.*0 %*	*16*.*2 %*	*5*.*8 %*	*24*.*4 %*	*33*.*1 %*	*0*.*5 %*	
2021	551.8	521.1	169.4	731.7	932.5	7.4	**2914**
	*18*.*9 %*	*17*.*9 %*	*5*.*8 %*	*25*.*1 %*	*32*.*0 %*	*0*.*3 %*	

From the tables produced for all member states, the year 2021 is highlighted in Table 5. The countries are listed by their two-letter country code, in the widespread protocol order used for EU countries. The sum of each row gives back 2021 data in Table 4, except for imports, because of intra-European imports originating from other member states.

**Table 5:** Gross electricity production [TWh] and percentages for each EU country for 2021 [[25], [26], [27]].

Country	CCGT	TPP	Biofuels	NPP	Renewables	Import
BE	22.5	3.5	4.7	50.3	19.0	0.0
	*22*.*5 %*	*3*.*5 %*	*4*.*7 %*	*50*.*3 %*	*19*.*0 %*	*0*.*0 %*
BG	3.0	17.4	2.6	16.5	8.0	0.0
	*6*.*4 %*	*36*.*7 %*	*5*.*4 %*	*34*.*7 %*	*16*.*8 %*	*0*.*0 %*
CZ	7.3	35.0	5.4	30.7	6.5	0.0
	*8*.*6 %*	*41*.*2 %*	*6*.*3 %*	*36*.*2 %*	*7*.*7 %*	*0*.*0 %*
DK	1.5	5.4	8.7	0.0	17.4	4.9
	*4*.*1 %*	*14*.*3 %*	*23*.*0 %*	*0*.*0 %*	*45*.*8 %*	*12*.*8 %*
DE	95.1	186.3	46.9	69.1	189.2	0.0
	*16*.*2 %*	*31*.*8 %*	*8*.*0 %*	*11*.*8 %*	*32.2 %*	*0*.*0 %*
EE	0.0	4.3	1.8	0.0	1.1	2.6
	*0*.*4 %*	*43*.*6 %*	*18*.*0 %*	*0*.*0 %*	*11*.*3 %*	*26*.*7 %*
IE	15.2	4.8	1.0	0.0	10.9	1.6
	*45*.*3 %*	*14*.*4 %*	*3*.*0 %*	*0*.*0 %*	*32*.*6 %*	*4*.*7 %*
EL	22.5	10.0	0.5	0.0	21.7	3.7
	*38*.*5 %*	*17*.*2 %*	*0.8 %*	*0*.*0 %*	*37*.*2 %*	*6*.*3 %*
ES	71.5	17.0	6.9	56.6	122.0	0.9
	*26*.*0 %*	*6*.*2 %*	*2*.*5 %*	*20*.*6 %*	*44*.*4 %*	*0*.*3 %*
FR	33.4	15.3	9.6	379.4	117.1	0.0
	*6*.*0 %*	*2*.*8 %*	*1*.*7 %*	*68*.*4 %*	*21*.*1 %*	*0*.*0 %*
HR	3.1	1.5	1.1	0.0	9.5	4.0
	*16*.*1 %*	*7*.*8 %*	*5*.*7 %*	*0*.*0 %*	*49*.*7 %*	*20*.*7 %*
IT	144.0	26.1	19.1	0.0	99.4	42.8
	*43*.*5 %*	*7*.*9 %*	*5.7 %*	*0*.*0 %*	*30*.*0 %*	*12*.*9 %*
CY	0.0	4.3	0.1	0.0	0.7	0.0
	*0*.*0 %*	*84*.*9 %*	*1*.*2 %*	*0*.*0 %*	*13.9 %*	*0*.*0 %*
LV	2.1	0.0	0.9	0.0	2.9	1.8
	*27*.*9 %*	*0*.*0 %*	*11*.*3 %*	*0*.*0 %*	*37*.*5 %*	*23*.*3 %*
LT	1.2	0.3	0.7	0.0	2.6	9.0
	*8*.*8 %*	*2*.*4 %*	*4*.*9 %*	*0*.*0 %*	*19*.*0 %*	*64*.*9 %*
LU	0.2	0.1	0.4	0.0	1.6	5.7
	*2*.*2 %*	*0*.*9 %*	*4*.*9 %*	*0*.*0 %*	*19*.*9 %*	*72*.*1 %*
HU	9.7	3.4	2.2	16.0	4.7	12.8
	*19*.*8 %*	*7*.*0 %*	*4*.*6 %*	*32*.*8 %*	*9*.*6 %*	*26*.*2 %*
MT	1.9	0.0	0.0	0.0	0.3	0.5
	*69*.*9 %*	*1*.*6 %*	*0*.*3 %*	*0*.*0 %*	*9*.*4 %*	*18*.*8 %*
NL	56.7	20.7	10.9	3.8	29.6	0.3
	*46*.*5 %*	*17.0 %*	*8*.*9 %*	*3*.*1 %*	*24*.*3 %*	*0*.*2 %*
AT	10.6	3.6	4.5	0.0	52.1	7.5
	*13*.*6 %*	*4*.*6 %*	*5*.*7 %*	*0*.*0 %*	*66*.*5 %*	*9*.*6 %*
PL	15.8	132.4	8.1	0.0	23.3	0.9
	*8*.*8 %*	*73*.*4 %*	*4.4 %*	*0*.*0 %*	*12*.*9 %*	*0*.*5 %*
PT	15.6	2.3	4.0	0.0	29.1	4.8
	*27*.*9 %*	*4*.*2 %*	*7*.*2 %*	*0*.*0 %*	*52*.*2 %*	*8*.*5 %*
RO	9.9	11.6	0.7	11.3	26.0	2.2
	*16*.*1 %*	*18*.*8 %*	*1.0 %*	*18*.*3 %*	*42*.*2 %*	*3*.*6 %*
SI	0.5	3.9	0.3	5.7	5.5	0.0
	*3*.*3 %*	*24*.*7 %*	*1*.*7 %*	*35*.*9 %*	*34*.*4 %*	*0*.*0 %*
SK	4.4	2.8	1.8	15.7	5.2	0.8
	*14*.*2 %*	*9*.*1 %*	*6*.*0 %*	*51*.*2 %*	*17*.*0 %*	*2*.*5 %*
FI	3.8	6.3	13.6	23.6	24.6	17.8
	*4*.*3 %*	*7*.*0 %*	*15.2 %*	*26*.*3 %*	*27*.*4 %*	*19*.*8 %*
SE	0.3	2.8	13.1	53.0	102.7	0.0
	*0*.*2 %*	*1*.*6 %*	*7*.*6 %*	*30*.*8 %*	*59*.*8 %*	*0*.*0 %*

BE: Belgium, BG: Bulgaria, CZ: Czechia, DK: Denmark, DE: Germany, EE: Estonia, IE: Ireland, EL: Greece, ES: Spain, FR: France, HR: Croatia, IT: Italy, CY: Cyprus, LV: Latvia, LT: Lithuania, LU: Luxemburg, HU: Hungary, MT: Malta, NL: Netherlands, AT: Austria, PL: Poland, PT: Portugal, RO: Romania, SI: Slovenia, SK: Slovakia, FI: Finland, SE: Sweden.

### 3.2 Total efficiency and energy-specific CO_2_ emissions

Based on the calculation method described in Chapter 2, the total efficiency (η_total_) and energy-specific CO_2_ emissions were computed. Table 6 shows the results for the EU-27.

**Table 6:** Total efficiency and energy-specific CO_2_ emissions for the EU-27.

Parameter	2011	2012	2013	2014	2015	2016	2017	2018	2019	2020	2021
η_total_ [−]	0.37	0.38	0.38	0.38	0.39	0.39	0.39	0.39	0.40	0.41	0.41
ε [gCO_2_/MJ]	113	109	103	98	100	100	101	96	89	82	84

**Fig. 3:**
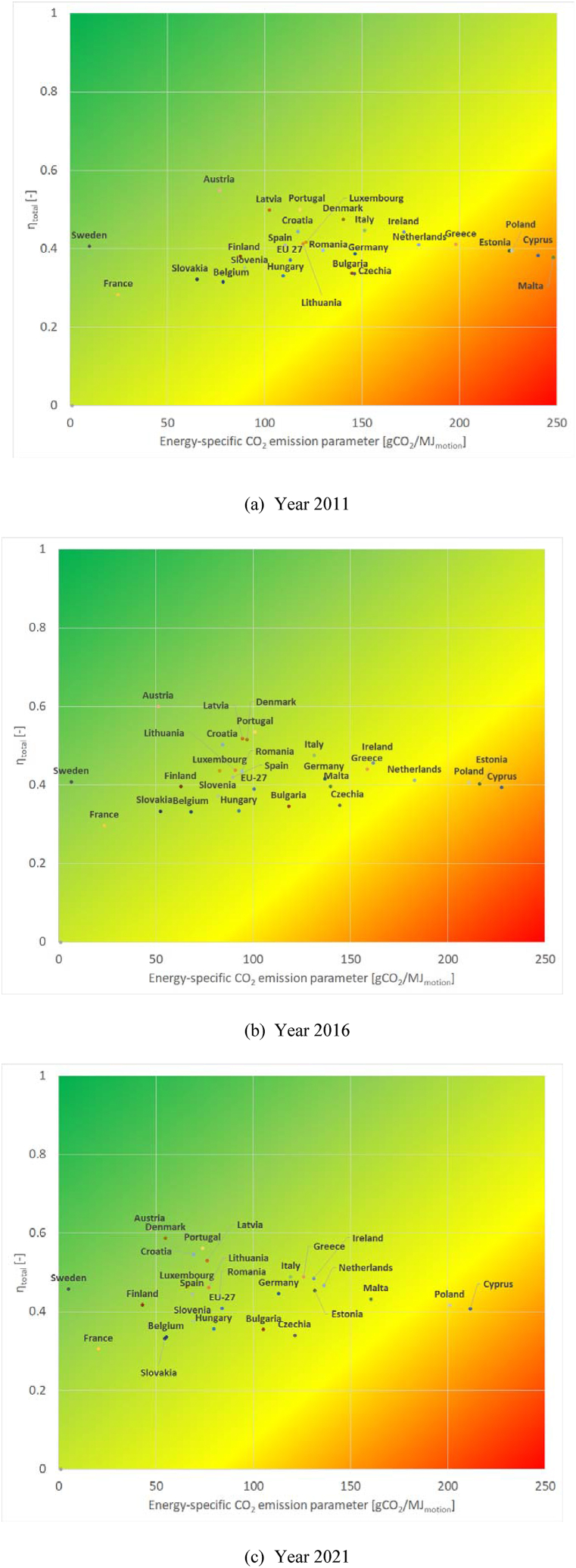
Sustainability matrices of all member states and the EU-27, drawn for 2011 (a), 2016 (b) and 2021 (c) (authors' creation).

Sustainability matrices [28] were drawn to better visualize the results. In the sustainability matrix, energy-specific CO_2_ emissions (ε) is placed on axis x, and total efficiency of energy conversion (η_total_) on axis y. The closer the results are to the top left corner, the higher the level of sustainability. This tool can well represent countries' progress towards more favorable stages in the examined period. Three years (2011, 2016, 2021) are highlighted in Fig. 3.

It is clearly visible from Fig. 3 that there were significant changes in the electricity mix in most countries during the observed period. However, there are still big differences between member states. According to the results, the two endpoints of 2021 from the ε parameter were Sweden (5 gCO_2_/MJ_motion_) and Cyprus (212 gCO_2_/MJ_motion_). Based on the total efficiency of energy conversion, Austria (with 0.61) is ranked first, while France (0.31) has the lowest value. Both parameters improved in all countries during the observed period, but their magnitude varied greatly. In the case of η_total_ the smallest percentage increase was found in Czechia (+1.0 %; +0.003), while Denmark had the biggest (+23.6 %; +0.11). The decrease in terms of the ε was always greater than 10 %, being the smallest in Poland (−11.4 %; −26 gCO_2_/MJ_motion_) and the highest in Denmark (−60.9 %; −85 gCO_2_/MJ_motion_). Therefore, in both parameters, Denmark improved the most in the observed period, but regarding energy-specific CO_2_ emissions, Finland (−50.8 %; −44 gCO_2_/MJ_motion_) and Sweden (−48.7 %; −5 gCO_2_/M_motion_J) can also be highlighted. In absolute value, the largest decrease in ε was achieved by Estonia (−41.7 %; −94 gCO_2_/MJ_motion_) and the smallest by France (−17.5 %; −4 gCO_2_/MJ_motion_). Besides Denmark, a high increase (over 20 %) in total efficiency was identified in Croatia (+22.7 %; +0.10).

In the case of Denmark, the improvement was due to the decline in CCGT (from 16.0 % to 4.1 % between 2011 and 2021) and TPP (from 41.6 % to 14.3 %), and the simultaneous steady increase in biofuels (from 12.0 % to 23.0 %) and renewables (from 26.8 % to 45.8 %), accompanied by an increase in the share of imports (from 3.6 % to 12.8 %).

**Fig. 4:**
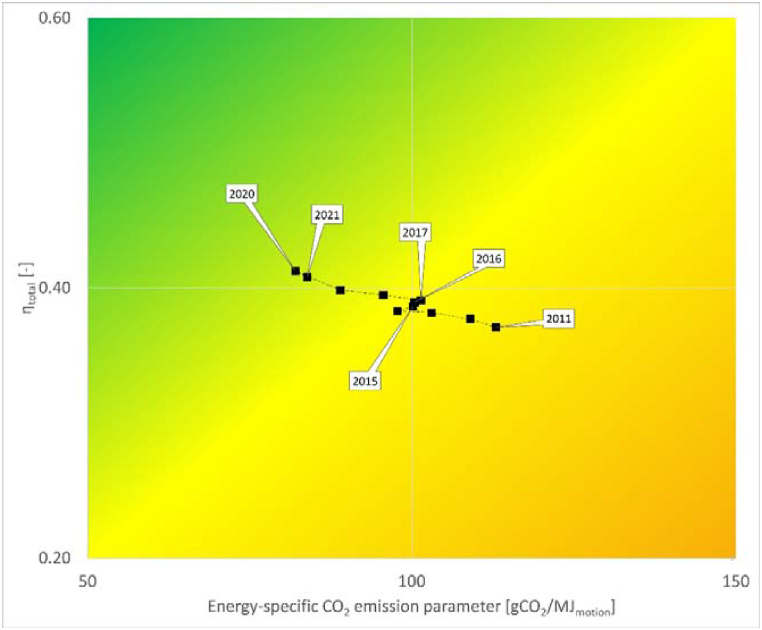
Changes in the two calculated parameters from 2011 until 2021 in the case of the EU-27 (zoomed in sustainability matrix) (authors' creation).

The EU-27 also achieved significant progress, moving from η_total_ = 0.37; ε = 113 gCO_2_/MJ_motion_ in 2011 and reaching η_total_ = 0.41; ε = 84 gCO_2_/MJ_motion_ in 2021. Fig. 4 shows the changes in the case of the EU-27. The total efficiency of energy conversion (η_total_) increased continuously, while the decrease in the energy-specific CO_2_ emissions (ε) was interrupted between 2014 and 2017. This interruption was mostly caused by the stagnating proportion of renewables and biofuels, paralleled by a significant increase in the proportion of natural gas in electricity production (see Table 4). This was largely due to market price changes, as during this period natural gas prices fell significantly, making CCGT plants more economical. 2021 also brought less favorable values from the sustainability point of view compared to 2020. In 2021, the proportion of renewables slightly decreased in addition to the increase in TPP. Although the production from renewable sources increased (+7 TWh compared to 2020), the far greater increase in total production (+115 TWh) was very significantly covered by thermal power plants (+69 TWh, see Table 4) due to market price changes, resulting in less favorable overall values.

**Fig. 5:**
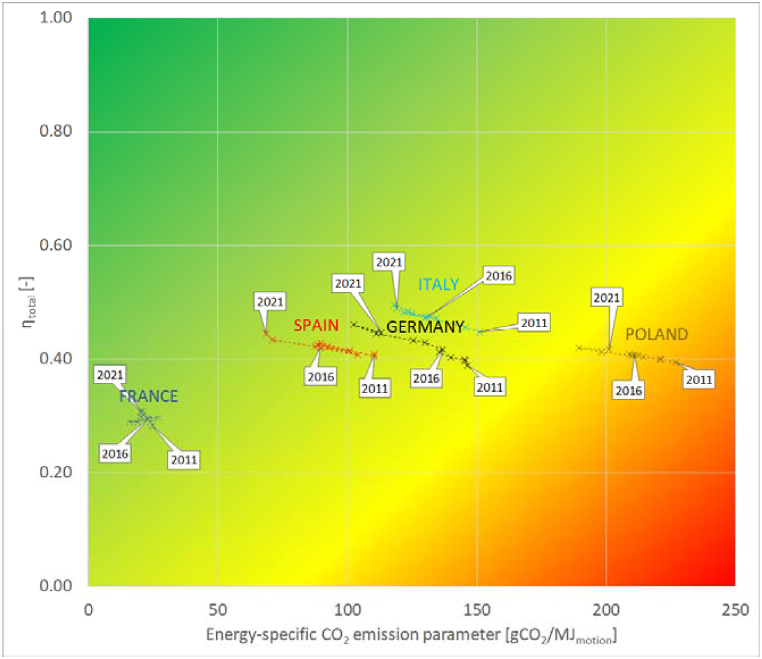
Sustainability matrix of the five most populous EU countries between 2011 and 2021 (authors' creation).

The population of the EU-27 in 2023 is around 450 million [34] inhabitants. The five most populous countries (Germany, France, Italy, Spain, and Poland) account for nearly two-thirds of the total EU population, therefore their role in the EU-27 average is dominant. The changes in the observed period for these countries are visualized in Fig. 5. The five countries are in very different parts of the matrix. France had the smallest energy-specific CO_2_ emission values, but the total efficiency was also the smallest due to the high ratio of nuclear power plants. Italy had the highest efficiencies but with medium ε values. Poland was behind the others in terms of the energy-specific CO_2_ emission parameter because of the large number of TPPs.

In all major countries, a very significant development can be observed, resulting in a displacement towards the upper left area of the matrix. The deterioration after 2020 is observable in Germany, Italy, and Poland, while further development can be seen in the case of France and Spain. The deterioration for 2021 in the case of Germany was mostly due to the decrease in the share of renewables (from 36.1 % in 2020 to 32.3 % in 2021) and the increase in the proportion of TPP (from 27.0 % to 31.8 %). In Italy, there was also a decrease related to renewables (from 31.8 % to 30.0 %) combined with an increase in import (from 10.3 % to 12.9 %) and CCGT (from 42.8 % to 43.5 %) percentages. Poland kept the renewables increasing (from 12.1 % to 12.9 %), but with a significant restoration of the thermal power plant proportion (from 65.2 % to 73.4 %), leading to worse parameter values.

### 3.3 Discussion

The fact that the source of electricity used for charging BEVs strongly determines the related emissions is well known. Among many other factors, coal-based electricity generation increases the specific emission values, while renewables lower such parameters. Compared to internal combustion engine vehicles, battery electric vehicle usage in the EU already meant around 50 % less emissions, according to mid-2010s data [11,35]. The calculation presented here led to similar results. Comparing the energy-specific CO_2_ emissions of gasoline and diesel engine vehicles (195 gCO_2_/MJ_motion_ [28]) to the calculated EU-27 ε value of 2015 (100 gCO_2_/MJ_motion_, see in Fig. 4) a coincidental ratio can be observed. This further improved in favor of BEVs as the ε value decreased to 84 gCO_2_/MJ_motion_ by 2021.

Rovai et al. [12] recently published gCO_2_e/MJ values for the EU. According to their WTW analyses, they obtained 96, 95 and 90 gCO_2_e/MJ for the years 2016, 2017 and 2018, respectively. Our methodology obtained similar results for the same three years (100, 101, 96 gCO_2_/MJ_motion_ orderly). Jochem et al. [16] assessed the CO_2_ emissions of electric vehicles in Germany, and they forecasted 0.38 kgCO_2_/kWh_el_ for 2020, which equals 106 gCO_2_/MJ_motion_ if 0.2 kWh/km BEV electricity efficiency is assumed [36]. This result is very close to the value (103 gCO_2_/MJ_motion_) determined here for the same country and year. Jochem et al. also made a forecast for Germany in 2030 with various scenarios, but in most cases the result was close to 0.3 kgCO_2_/kWh_el_. With the same efficiency assumption, this equals 83 gCO_2_/MJ_motion,_ and it is worth noting that it is already very close to the value of 84 gCO_2_/MJ_motion_ calculated here for the EU-27 (2021). This means that Germany is projected to reach the current EU-27 level around 2030 in terms of energy-specific CO_2_ emissions.

It is evident that the average CO_2_ intensity of electricity generation is declining, not just in Europe but globally. According to the International Energy Agency [18], the 459 gCO_2_/kWh average intensity for 2021 will decline to 165–330 gCO_2_/kWh by 2030 according to various scenarios. The current 459 gCO_2_/kWh means 169 gCO_2_/MJ for BEVs, based on the same assumptions and efficiencies presented in this article. The calculated EU-27 (2021) value of 84 gCO_2_/MJ is less than half of the global average, reflecting the efforts already made by EU members to decarbonize. However, big differences between EU countries are well known [37] and are also visible in the sustainability matrices drawn here (Fig. 3). Compared to the 169 gCO_2_/MJ world average value, it can be concluded that only two member states (Poland and Cyprus) exceed this level.

As electric vehicles are on a continuous and rapid rise, questions regarding the sustainability of their usage are gaining more attention. After China, Europe has the second-largest electric vehicle stock, with an impressive growth rate of 64.3 % in 2021 [38] and 15 % in 2022 [39]. The growth is projected to be exponential in the near future [[39], [40], [41]], giving a rapidly increasing proportion of BEVs among all passenger cars. This will lead to important questions not only related to emissions but also regarding electricity needs, which can be relevant to future research directions, too.

This paper evaluates the battery electric vehicle usage in the EU from a sustainability perspective based on two parameters (total efficiency of energy conversions and energy-specific CO_2_ emissions), which obviously leads to certain limitations. It cannot cover all aspects of sustainability, and the WTW method also has its limitations. Further extension of the investigation to full LCA would also have practical benefits, making it a promising future research direction.

## 4 Conclusions

This article evaluated BEV usage in EU countries from 2011 until 2021 from a sustainability-based perspective. The calculations aimed to define two parameters: the energy-specific CO_2_ emissions (ε) and the total efficiency of energy conversions (η_total_). An important basis of the calculation was the electricity mix of each country, which typically went through significant changes during the period. Sustainability matrices were drawn to track the changes and present the results for the observed period. There are relevant differences between the member states, but in general, the EU-27 achieved a 4 % increase in total efficiency and a 29 gCO_2_/MJ_motion_ decrease (−26 %) in 11 years.

One notable theoretical benefit of this work is to present an approach which evaluates the BEV usage in all member states by the same complex parameters using a common dataset. Therefore, the results are strongly comparable, resulting in a good basis of assessment. The different policies and pathways of the member countries become highly visible and assessable, from which not just the member states can learn and move their transportation sector in a more sustainable direction. Country-level evaluations can help the member states to direct their actions towards required fields.

Aspirational goals have already been set, and to reach them, tracking and assessing BEV usage in terms of its environmental impacts remains an important issue. Ambitious policies are projecting rapid and massive changes regarding the transportation sector. Further research should continue the evaluation of electric vehicles from a sustainability perspective. To meet future needs, a complex evaluation of the electricity and transport sector is of great importance, making it a priority research topic. Without knowing the environmental and economic effects of electromobility long-term decision-making is becoming very vulnerable. The aim of this work was to contribute to making the picture clearer, although it has limitations that should be mentioned. Here, sustainability was described with the help of two parameters (the total efficiency of energy conversions and the energy-specific CO_2_ emissions), which are clearly not capable of covering all its aspects. But based on the authors’ opinion they well represent BEV usage, and progress in any of them unequivocally points towards sustainability. Therefore, this makes these two parameters an adequate way to evaluate the development towards sustainability.

## Data availability statement

Data associated with this research has been deposited into a publicly available repository: Kocsis, Denes (2024), “Data for BEV usage in the EU (member states energy mix)”, Mendeley Data, V2, https://doi.org/10.17632/5tzpxx5w8x.2.

## CRediT authorship contribution statement

**Denes Kocsis:** Writing – review & editing, Writing – original draft, Visualization, Investigation, Formal analysis, Conceptualization. **Judit T. Kiss:** Writing – review & editing, Validation, Supervision, Investigation. **Istvan W. Arpad:** Writing – review & editing, Supervision, Methodology, Investigation, Formal analysis, Conceptualization.

## Declaration of competing interest

The authors declare that they have no known competing financial interests or personal relationships that could have appeared to influence the work reported in this paper.
